# Supplementary figures and images for: Infection of Plasmodiophora brassicae changes the fungal endophyte community of tumourous stem mustard roots as revealed by high-throughput sequencing and culture-dependent methods
Source: PLoS One. 2019 Jun 12;14(6):e0214975. doi: 10.1371/journal.pone.0214975 (PMC6561537; doi:10.1371/journal.pone.0214975)

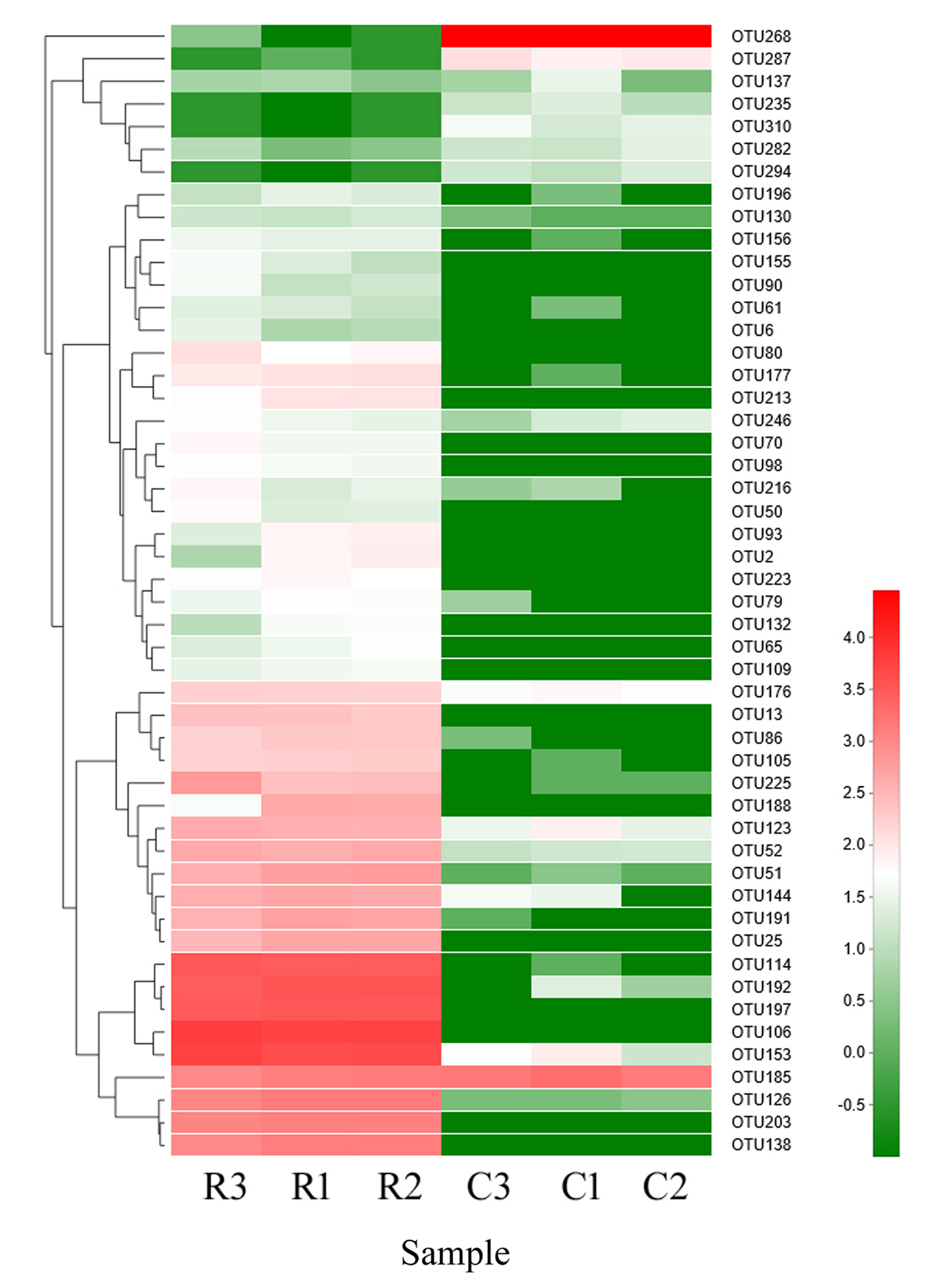

Supplement: S1 Fig — R, healthy roots; C, clubroots. (JPG) [file pone.0214975.s001.jpg]

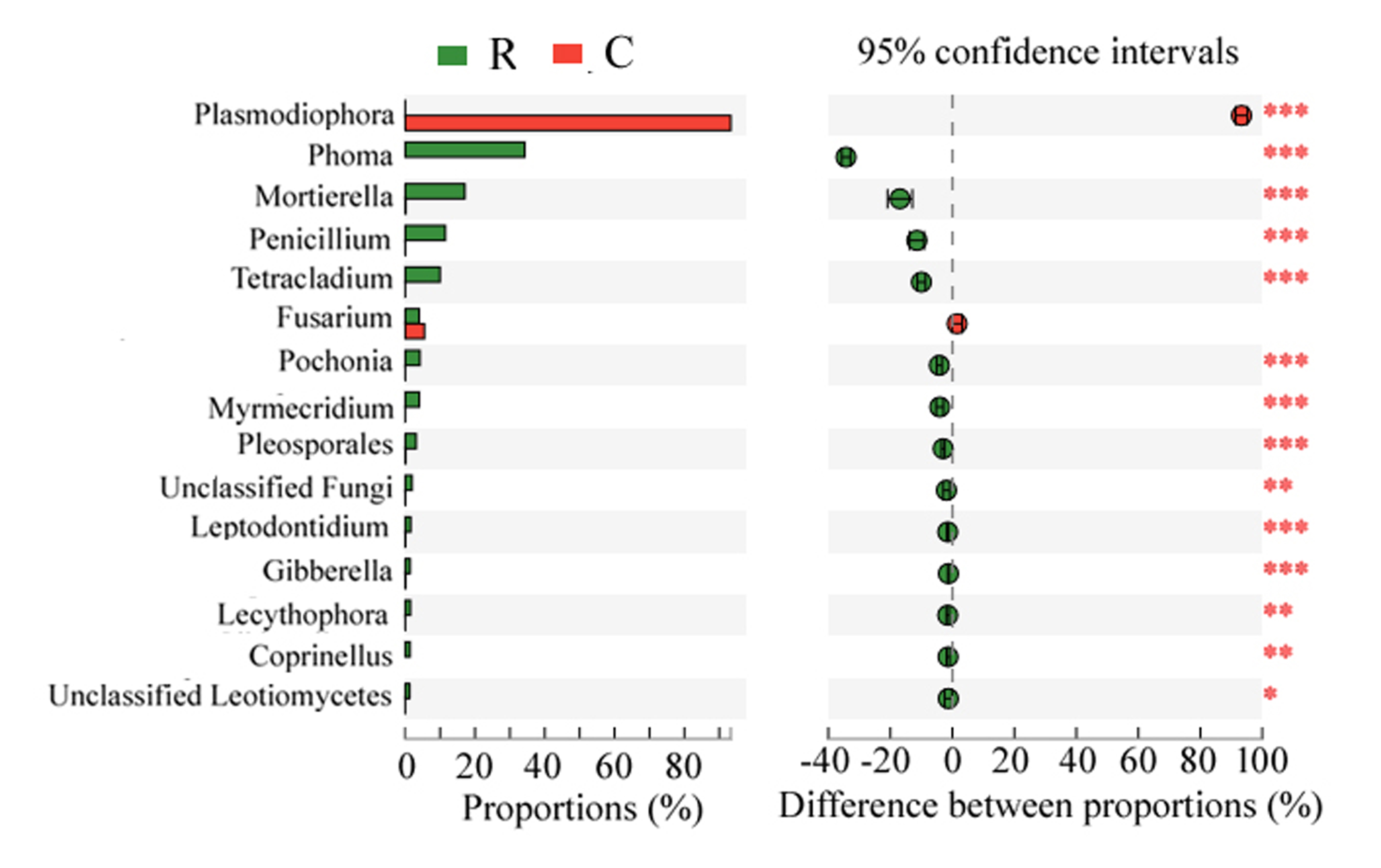

Supplement: S2 Fig — p<0.05*, p<0.01**, p<0.001***. R, healthy roots. C, clubroots. (JPG) [file pone.0214975.s002.jpg]
